# Meteorological Register for March, 1841

**Published:** 1841-04-10

**Authors:** J. Conrad


### METEOROLOGICAL REGISTER FOR MARCH, 1841.

*Kept at the Pennsylvania Hospital, by J. Conrad.*

| Date. | Thermometer. Max. | Min. | 9 AM. | Barometer. 10 AM | 3 PM. | Dew Point | Winds. Direction. | Force | Rain. | REMARKS. |
|---|---|---|---|---|---|---|---|---|---|---|
| 1 | 55 | 32 | 42 | 29.94 | 29.92 | 31 | SW.W. | 1 | | Morning partly clear; afternoon clear. |
| 2 | 56 | 33 | 44 | 30.04 | 30.00 | 30 | NE. | 0 | | Fair, but hazy. |
| 3 | 51 | 36 | 40 | 29.88 | 29.83 | 32 | NE. | 1 | | Clear from 10 AM. |
| 4 | 44 | 32 | 37 | 30.10 | 30.10 | 13 | N.NW. | 1 | | Clear. |
| 5 | 32 | 22 | 24 | 30.49 | 30.44 | 1 | NE. | 1 | | Clear; cloudy from 4 PM. |
| 6 | 46 | 26 | 27 | 30.16 | 29.82 | 34 | NE. | 4 | 1.904 | *Storm; snow all morn; rain in aft'n and ev'g. |
| 7 | 49 | 35 | 41 | 29.61 | 29.58 | 33 | W.NE. | 2 | .288 | Morning clear; rain from 3½ PM. all evening. |
| 8 | 43 | 34 | 40 | 29.79 | 29.85 | 26 | NW. | 3 | | Cloudy; snow squall at 9 AM. |
| 9 | 43 | 34 | 37 | 30.25 | 30.28 | 21 | NW. | 3 | | Morning clear; aft'n and ev'g partly clear. |
| 10 | 37 | 32 | 36 | 30.21 | 30.09 | 31 | NE. | 3 | .500 | Snow all day from 7½ AM. |
| 11 | 42 | 32 | 36 | 30.14 | 30.16 | 23 | NW. | 1 | | Clear. |
| 12 | 35 | 27 | 32 | 30.30 | 30.12 | 27 | NE.E. | 2-4 | 1.210 | Morn cloudy; sleet in aft; rain in eve'g, blowing a gale. |
| 13 | 45 | 33 | 38 | 29.44 | 29.45 | 30 | WSW. | 3 | | Morning partly clear; cloudy from 3 PM. |
| 14 | 46 | 28 | 39 | 29.87 | 29.85 | 21 | NW. | 2-5 | .025 | †Morn partly clear; aft. cloudy; rain & snow at dusk; gale in evg |
| 15 | 35 | 22 | 26 | 30.27 | 30.20 | 14 | NW.SE. | 0 | | Morning clear; afternoon cloudy; snow at 10 PM. |
| 16 | 32 | 24 | 31 | 30.05 | 30.13 | 22 | NE. | 4 | .500 | Snow till 5 PM; therm'r, at 2 PM, 26°. |
| 17 | 31 | 21 | 27 | 30.37 | 30.32 | 10 | NE. | 4 | | Cloudy; 5 or 6 inches of snow on the ground. |
| 18 | 41 | 28 | 33 | 30.03 | 29.96 | 23 | NE. | 2 | .696 | Sleet from 6½ AM; rain in aft'n till 7 PM. |
| 19 | 56 | 36 | 45 | 30.14 | 30.13 | 22 | NW.SW. | 1 | | Clear. |
| 20 | 68 | 35 | 45 | 30.11 | 30.04 | 35 | SW. | 1 | | Fair but hazy; even'g cloudy, with sprinkling of rain. |
| 21 | 58 | 41 | 52 | 30.20 | 30.18 | 24 | NW. | 2 | | Clear. |
| 22 | 53 | 34 | 40 | 30.36 | 30.24 | 30 | NE.SE. | 2 | | Morn fair, but hazy; aft cl'dy. |
| 23 | 63 | 40 | 59 | 29.82 | 29.67 | 46 | S.NW. | 3 | .327 | Morn cloudy; rain from 1½ till 5½ PM; evg clear and calm. |
| 24 | 59 | 34 | 46 | 30.02 | 30.00 | 37 | SW. | 1 | | Cloudless. |
| 25 | 65 | 38 | 51 | 30.18 | 30.12 | 37 | SW. | 2 | | Clear. |
| 26 | 70 | 41 | 55 | 30.13 | 30.04 | 44 | SW. | 2 | | Fair, hazy. |
| 27 | 72 | 49 | 59 | 30.02 | 29.97 | 52 | SW. | 1 | | Fair, hazy. |
| 28 | 71 | 51 | 59 | 29.88 | 29.88 | 48 | NE. | 1 | | Partly clear; hazy; rain in eve. |
| 29 | 48 | 41 | 43 | 29.92 | 29.67 | 40 | ENE. | 2 | .370 | Light rain. |
| 30 | 45 | 34 | 45 | 29.89 | 30.00 | 28 | N.NE. | 2 | | Cloudy. |
| 31 | 49 | 26 | 34 | 30.36 | 30.23 | 26 | NE.SW. | 2 | | Clear; evening cloudy. |
| Mean | 49.67 | 33.26 | 40.74 | 30.06 | 30.00 | 28.66 | | | 5.821 | |

| | | | |
|---|---|---|---|
| Mean temperature, | 41.22° | Clear days, - | 15 |
| " pressure, | 30.04 inches. | Clouds, rain, snow, &c. - | 16 |
| " dew-point, | 28.66° | | |

*Winds*—W. to S. 8 days; S. to E. 3 days; E. to N. 12 days; N. to W. 8 days.

* Barometer fell to 29.27 at 10 P. M.; the maximum of thermometer occurred in evening. † Aurora.

