# Errata

**Published:** 1841-05-15

**Authors:** 


ERRATA.—The following omissions occurred in the column for rain in the Meteorological Register for April :—

.328 on the 10th.
.010 on the 24th.

